# Numerical and Experimental Analysis of Matched Filter Interrogation of FBG Sensors with Large Side Lobes

**DOI:** 10.3390/s20195522

**Published:** 2020-09-27

**Authors:** Krzysztof Skorupski, Sławomir Cięszczyk, Patryk Panas, Piotr Kisała

**Affiliations:** Department of Electronics and Information Technology, Lublin University of Technology, 20-618 Lublin, Poland; k.skorupski@pollub.pl (K.S.); s.cieszczyk@pollub.pl (S.C.); p.panas@pollub.pl (P.P.)

**Keywords:** FBG sensors, interrogation, inverse apodization

## Abstract

This article presents the effect of fiber Bragg gratings side lobes on interrogation systems consisting of sensor and matched filters. The conducted research shows that high-value side lobe structures applied as sensors and/or filters are characterized by some interesting properties. The paper presents both numerical analysis and experimental verification of the fiber Bragg gratings (FBG) interrogation systems with matched filters for gratings containing high side lobes. Numerical modeling of Bragg structures was performed for two different cases: uniform and inverse apodization. Modification of apodization can change the side lobe reflectance level even above levels found in uniform structures. This is a case not described in the literature, especially in terms of possible applications. Transfer characteristics, i.e., the relationship between power intensity as a function of wavelength shift, were determined. A collection of gratings with spectra corresponding to those analyzed in numerical experiments were fabricated. Next, the transfer characteristics of the interrogation systems containing real FBG were determined. The properties of the proposed systems are described. It has been shown that a significant level of sidebands, which is often the subject of many drawbacks in filtering or telecommunications systems, can be an advantage. It has been demonstrated that a high level of side lobes can be used to increase the measurement range of the FBG sensor interrogation systems. It has been determined numerically and confirmed experimentally that from the point of view of the design of sensor interrogation systems, it is beneficial to combine specific pairs of gratings: one with a spectrum characterized by a low side lobe level and a second one in which the spectrum has very high side lobes.

## 1. Introduction

**Figure 1:**
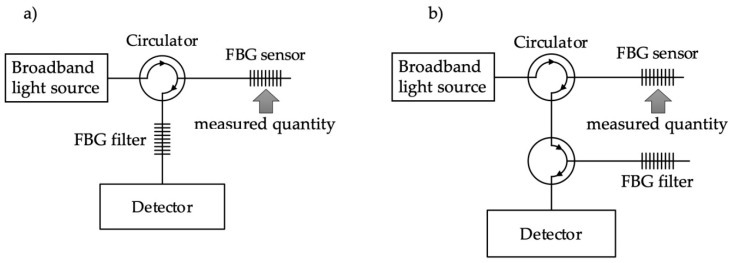
Basic configurations of interrogation systems: (**a**) transmissive; (**b**) reflective.

The advantages of sensors with fiber Bragg gratings (FBGs) have led to their high popularity and extended their field of application [1,2,3,4,5,6,7]. These advantages can be divided into two groups. The first group comprises properties of all fiber optic sensors, such as small size, weight, and the possibility of remote measurements [8]. The second group results from wavelength modulation of light that is reflected or passed through the grating (Figure 1). The spectral transduction property of FBG sensors means that changes in the measured quantity, such as elongation and temperature, are converted into a wavelength shift. Application of an appropriate demodulation method, in turn, is required to change the wavelength shift to radiation intensity. Many interrogation schemes have been proposed [9], including portable energy-efficient interrogators based on the spectral convolution between the sensors and tunable filters [10]. Recently, the method based on electrical harmonic analysis have been also discussed, but it requires a harmonic modulator and appropriate signal acquisition system [11]. So, it is a bit more complicated solution. Moreover, this particular mathematical model is dedicated to long period grating (LPG) interrogation. Additional features of the interrogation method can be insensitivity to changes in the light source intensity as well as insensitivity to changes in attenuation along the entire optical path. An advantage is also the possibility of having multiple FBGs sensors (differing in wavelength) on one transmission path. Changes in a physical quantity, such as elongation, force, or temperature, lead to modification of the grating parameters—mainly its period—and thus the spectral shift of its spectral characteristics. In the process of measuring a specified physical quantity, the Bragg wavelength shift should be determined. It can be done in several different ways. The basic method is to use a broadband light source and optical spectrum analyzer (OSA). This method is most often used at the stage of developing a specific sensor or in laboratory tests. Optical spectrum analyzers allow measurements to be taken with very high resolution; however, they are very expensive and are rather non-portable devices due to their significant weight and size. Other popular demodulation methods using broadband light sources include interferometric techniques and methods using optical filters. Examples include a Fabry–Perot interferometer [12] as well as edge filters with different spectral characteristics [13]. To transform the FBG spectrum shift into photodetector power, an optical element whose spectral characteristics depend monotonically on the wavelength can be used. The simplest component of this type is a radiation source. However, this solution is not flexible because the spectral characteristics of the light source cannot be arbitrarily shaped. Many more options and possibilities for shaping and selecting transfer characteristics of the sensor system can be achieved with various types of optical filters. There are two types of filters: one with a spectrum much wider than that of the sensor and another with spectrum width similar to the spectrum of the sensor. They are called wideband edge filters and matched filters [14,15] and have spectra similar to that of the sensor itself. Further improvement of the properties of such systems can be obtained through the use of a parallel matching technique [16,17]. In the second example, properly selected FBG periodic structures are used. This solution is relatively cheap and provides appropriate sensor parameters such as sensitivity and linearity. It is possible here to use two main configurations in which the power transmitted (Figure 1a) or reflected (Figure 1b) through the sensor grating is measured by the photodetector. Depending on the shapes of the spectra of both gratings used in the transmissive and reflective systems, slightly different properties can be obtained (Figure 1). Systems with matched filter interrogation have been considered in the literature in various aspects, e.g., in terms of linearity or speed of operation [16]. When considering the shapes of sensor transfer characteristics, the most important task of an optical filter system is to achieve proper linearity or to increase the dynamic range of the system [18]. It seems that systems containing a matched filter can be adapted to applications; moreover, they allow the measurement range to be selected through their spectra width. They are also extremely fast and are characterized by good accuracy. In some cases, a piezoelectric transducer (PZT) can be used to tune the wavelength of the filtering gratings [19]. It should be noted, however, that compared to the static system, this causes both system complications and a significant increase in costs.

It must be emphasized that literature reports focus clearly on Gaussian-shaped sensors and filters. This is due to the fact that such analysis is relatively simple and can even obtain analytical formulas of relation of optical power versus Bragg wavelength shifts [6]. For other shapes, comparisons can be made only using numerical methods. Gratings with a Gaussian spectra shape are apodised structures; thus, they have no side lobes in the spectrum. Creating FBG with a perfectly Gaussian shape and, at the same time, a high reflectance value is a difficult task. An ideal gaussian shape can only be achieved for weak structures with low reflectance. Overall, if a higher reflectance is obtained, then more power can reach the detector, and thus a greater dynamic range is achieved. Dynamic range and system linearity depend on the full width at half-maximum (FWHM) of the FBG structure. Widening of the FWHM parameter of a gaussian FBG is unfortunately often associated with a decrease in its reflectance. Accurate analyses and formulas for the power obtained on the detector depending on the Brag wavelength shift can be found in several publications [18]. The second type of grating proposed in the literature is the matched-filter interrogation system with a rectangular spectrum shape. This solution achieves a linear relationship between the power on the detector and the change in the measured physical quantity. The rectangular shape of the spectrum can be achieved using chirped fiber Bragg gratings (CFBGs) [20].

In general, the transfer characteristics of matched filter interrogation systems consist of two regions. The first is the linear region characterized by high sensitivity. The second region is placed in the wavelength range near the Bragg wavelength. This part is called the Bragg region, and in strong gratings it is flattened due to the saturation of spectral characteristics. For both wide-edge filters and matched filters, it is possible to model the spectrum edge characteristics by using chirp and apodization parameters [21]. Shaping the conversion between the change in wavelength and intensity by matching the shapes of spectra allows one to obtain the right solution for the specific application with the required measurement range. For the edge filters, the shape of only one filter edge can be chosen, while for the matched filter system the shapes of both structures should be chosen for the sensor and filter.

There are two basic types of interrogation systems using FBG. Figure 1 shows the diagrams of transmissive and reflective modes.

In the case of the system shown in Figure 1a, a significant drawback is that the detector measures the optical power of the signal transmitted through the sensing structure. As a result, it is necessary to divide the sensor head itself and provide two fibers: one supplies the radiation that illuminates the sensor, and the second transmits the signal from the sensor to the detector. The systems presented in Figure 1b do not have this drawback, offering so-called single-end operation, where the signal reflected from the grating operating as the transducer of the measured quantity is taken into account.

In this article, we try to answer the research question about the impact of side lobes of Bragg gratings on matched filter interrogation method in reflection mode. We analyze properties that can be obtained using structures with a high side lobe. The basic issue is the question of how side lobes affect the transfer characteristics of the measurement system. We investigate the possibility of shaping such characteristics and increasing the measurement range. Additionally, we study a specifically selected apodization method (inverse apodization) to achieve side lobes with significant reflectivity and determine their influence on the properties of the interrogation system [22,23].

## 2. Modeling Spectra of Fiber Bragg Gratings with High-Reflectivity Side Lobes

**Figure 2:**
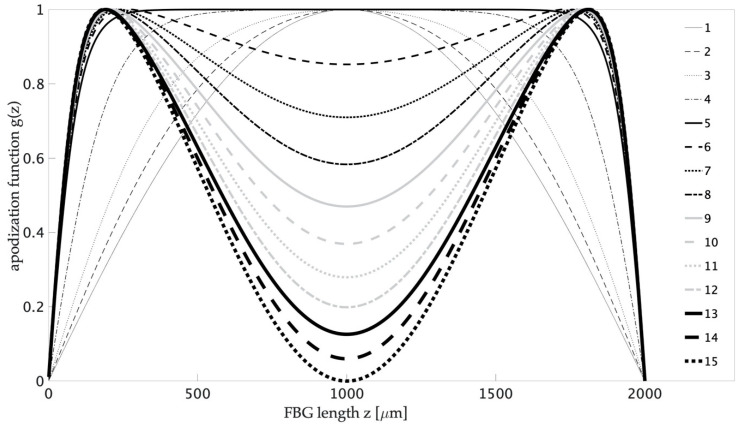
Apodization profiles used in numerical modeling of fiber Bragg gratings (FBG) spectral characteristics.

A significant level of side lobes in spectra characterizes uniform FBGs without intended apodization. It should be added that, in most applications, this is an undesirable property. The appearance of the side lobes distorts the spectral characteristics of the FBG. In the case of their use, e.g., in telecommunications, it causes distortions of signals that are subject to such filtering. To determine the impact of different side lobe levels on interrogation systems, firstly, the method to create structures with such properties should be determined. Numerical simulations and mathematical models for FGB spectra were discussed comprehensively [24,25]. Numerical calculations were carried out in OptiGrating software, as a result of which FBG spectral characteristics were obtained. The structural parameters of the optical fiber corresponding to SMF-28e+ have been introduced into the model. The length and depth of the modulation of the refractive index of gratings were selected in such a way that the obtained spectra were characterized by the same reflectance and main-lobe FWHM value. Various apodization profiles were calculated, resulting in the shape of the spectra having different side lobe reflectance values. Thus, it was possible to compare FBG interrogation systems with different side lobe strengths. The parameter that affects side lobe reflectance is the axial apodization of the Bragg structure [1,26]. Figure 2 presents the apodization profiles used in the calculations. Numerical calculations were performed for structures with a length of 2 mm. The apodization function g(z) was described by the following equation:

(1) g(z)=a tgh(z)⋅a tgh(1−z)⋅(b cos(2πz)+1),

where *a* and *b* are constants and are both parameters of fiber Bragg gratings calculated for analysis with values listed in Table 1.

The refractive index modulation was changed in the range from 2 to 4 × 10^−5^ and that the reflective index R was equal for all structures. The solid lines mark three reference profile shapes: Gaussian, uniform, and fully inverted. Intermediate profiles are marked with dashed lines.

**Figure 3:**
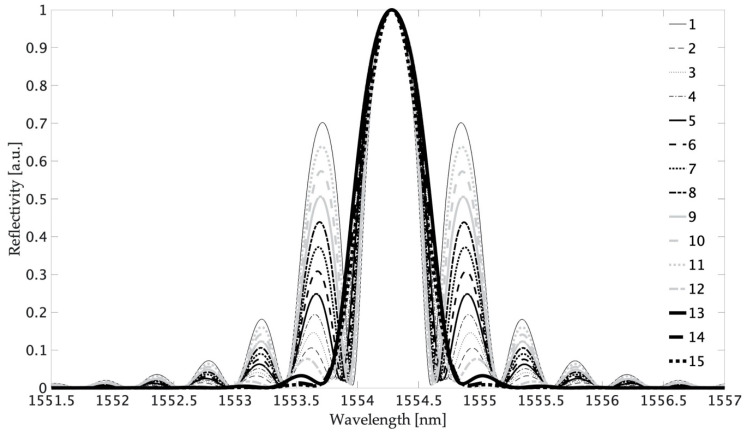
Spectra of the fiber Bragg gratings for apodization profiles from Figure 1.

Figure 3 shows reflection spectra of modeled FBG. The reflectance of all structures was equal to 1, and the FWHM parameter varied from 0.44 to 0.62 nm. To analyze the obtained results, the parameter *K* characterizing FBG spectra was used, defined as the ratio of the maximum reflection coefficient of the first-order side lobe to the reflectance of the main lobe. For the apodization profile similar to the Gaussian distribution, the *K* parameter was equal to 0. The shape of apodization close to the rectangle (uniform) gave side lobes with reflectivity of 0.08 and *K* = 0.1. Obtaining higher side lobe reflectance is possible through the use of inverted apodization profiles. The maximum calculated value of side lobe reflectance was 0.5318, and the coefficient *K* was equal to 0.7014.

**Figure 4:**
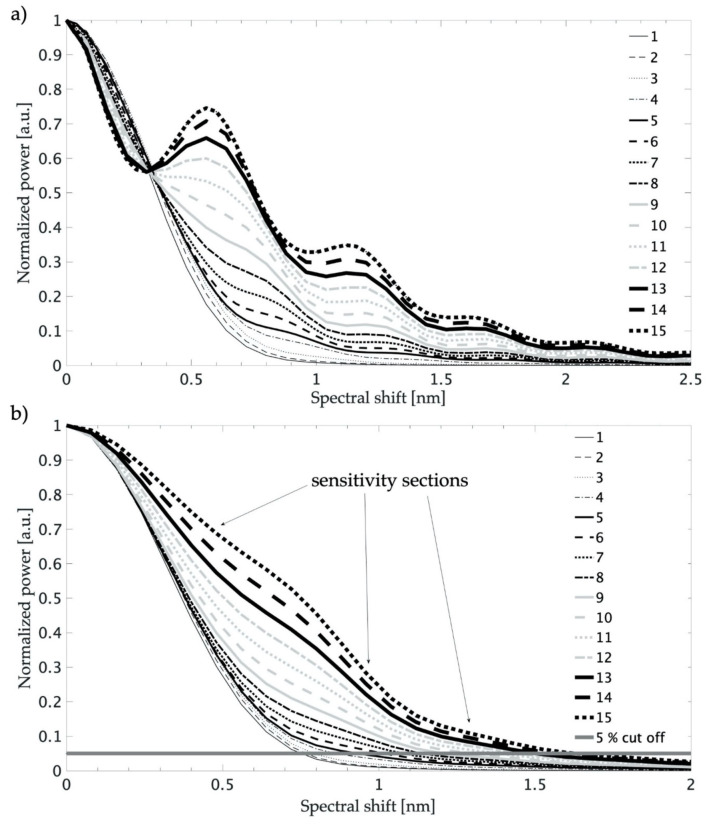
Comparison of the shape of the transfer characteristics of the matched filter system: (**a**) two similar structures; (**b**) uniform FBG1 with one of the FBG1–FBG15 filters.

The operation of interrogation systems with a matched filter was simulated. The calculations rely on two numerical operations with the spectra. First is the shift of spectral characteristics of one fiber Bragg grating (sensor) with the fixed (not shifted) spectrum of the other grating. Next, the spectra product corresponding to the common part of the characteristic is determined. Dependence of the spectra structure products as a displacement (spectral shift) function is equivalent to an optical filtration operation, for different grating pairs, which is shown in Figure 4. One of the parameters that can be used to analyze the results is the spectral shift range, in which the amplitude of the output signal is reduced to 5% of its maximum value. The calculations were carried out for two cases. In the first, the shapes of the two spectra (filter and sensor) were identical. Figure 4a shows the numerically determined characteristics of the interrogation system. Obtained curves had irregular shapes and were not monotonic. In the second proposed approach, the smallest side lobe spectrum was chosen as the reference (filter) structure, which corresponds to the grating with a Gaussian profile apodization. The spectra with non-zero side lobes and different reflection coefficients were selected as the sensor structure. In this case, the mutual spectral shift representing the measured range (5% of maximum intensity) had the smallest range of 0.753 nm. With higher side lobe levels, the width of the transfer characteristics increased and reached a value of 1.632 nanometers for the pair of simulated grating spectra 1 and 15.

The transfer characteristics of sensors with the interrogation system are presented in Figure 4b. Individual curves had varying slopes, and in some ranges the characteristics were linear. The slope of this curve determines the sensitivity of the interrogation system. The highest sensitivity was obtained for the system with two FBGs with the smallest side lobes (green line). This sensitivity was 1854 a.u. per nm. For the system with two different FBGs (one with and one without side lobes), two or three ranges of transfer characteristics for which the shape of the characteristic is close to linear can be distinguished. This property is important in many applications of FBG sensors in real measurement systems. The slopes of individual parts of the characteristic were different; thus, the sensitivity of the sensor system also varied. For FBG structures, for which the *K* factor was in the range from 0.077 to 0.572, the initial parts of the curves were characterized by a steeper slope; in the middle part, the slope had an intermediate value and was the smallest among the larger values in the spectral shift. The transfer characteristics obtained for *K* = 0.31 (blue line) reached a sensitivity of 1.515 a.u. per nm in the range from 0.16 to 0.48 nm, 0.416 a.u. per nm in the range from 0.64 to 1.04 nm, and 0.076 a.u. per nm in the range from 1.2 to 1.6 nm. For *K* = 0.64 (black line), the sensitivities of the two linear sections of the transfer characteristic were equal. In the 0.16 to 0.56 nm range, the sensitivity was 0.905 a.u. per nm, and for the range from 0.8 to 1.04 nm, the sensitivity was 0.909 a.u. per nm. In the remaining part in the range from 1.2 to 1.6 nm, the sensitivity was equal to 0.17 a.u. per nm. The transient characteristic for the FBG15 structure and *K* = 0.70 (red line) reached the highest sensitivity of 0.99 a.u. per nm in the central part of the characteristic in the range from 0.72 to 1.04 nm. In the range from 0.16 to 0.64 nm the slope was 0.755 a.u. per nm, and it was 0.194 a.u. per nm in the range of 1.2 to 1.6 nm.

Therefore, after analyzing the simulations, it can be assumed that it is more advantageous to combine pairs of gratings, in which the spectrum of the first is characterized by a low level of side lobe reflectance (preferably zero) and the second whose spectrum has a higher reflectance of side modes. These FGB pairs can extend and shape the slope of the transition characteristic. Pairs of structures with similarly high side lobe reflectivities have slightly different properties. Their transfer characteristics are slightly wider, which results in a larger measurement range, but the shape of the characteristic is irregular and difficult to model.

## 3. Experimental Verification

For experimental verification, a group of fiber Bragg gratings with different spectral characteristics was fabricated. Table 2 shows the parameters of the analyzed fiber Bragg gratings.

**Figure 5:**
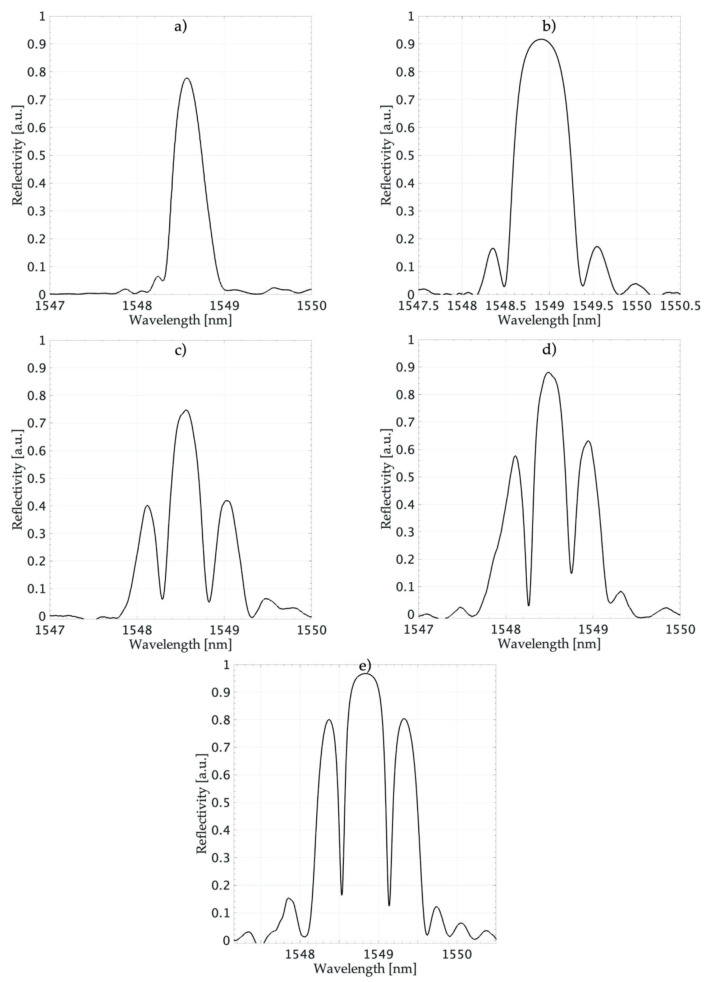
Spectral characteristics of fabricated FBGs: (**a**) FBG1; (**b**) FBG2; (**c**) FBG3; (**d**) FBG4; (**e**) FBG5.

FBG structures were fabricated using the phase mask method, which implemented a system of beam shutters that allows the apodization profile of the written structures to be controlled. The *K* factor varied from 0.0217 to 0.826. Figure 5 shows the spectral characteristics of written structures.

**Figure 6:**
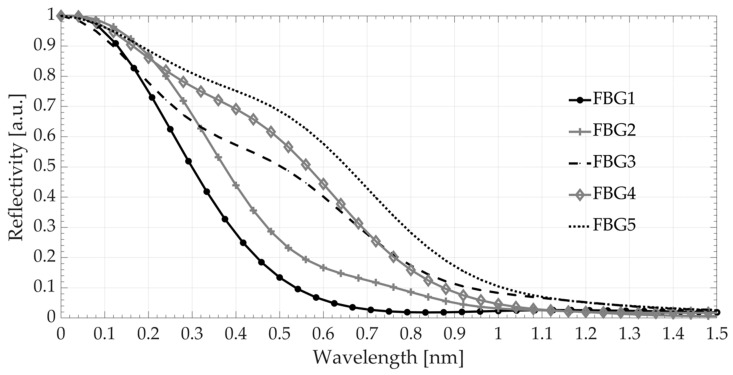
Calculated transfer characteristics based on the spectra of real structures.

The produced structures had different FWHM and reflectance. To compare the properties, the reflectance coefficient was normalized. Afterward, numerical calculations were performed in a manner corresponding to the calculations carried out for the modeled spectra. Figure 6 shows the calculated interrogation characteristics.

The shapes of the characteristics corresponded to the results obtained during numerical calculations. There were noticeable ranges of transfer characteristics with different slopes corresponding to the main Bragg peak and first side lobe amplitude. For pairs FBG1 with FBG4 and FBG5, the maximum sensitivity was obtained in the middle range of the transfer characteristics. For the pairs of FBG1 with FBG3 and FBG2 gratings, the largest slope of the characteristic was obtained in the initial range. Then, the sensitivity decreased in the middle of the transient characteristic and was further reduced in the final part. Considering the 5% cut-off level of the characteristic, the measurement range doubled, from 0.62 to 1.22 nm.

**Figure 7:**
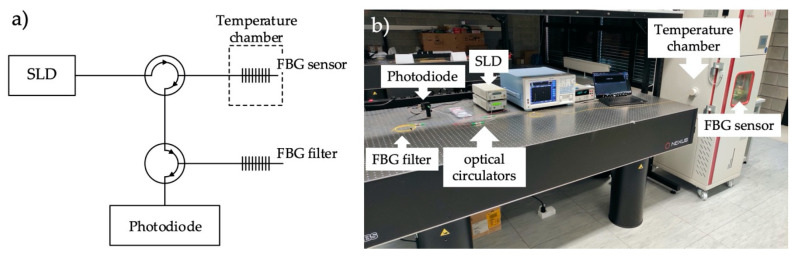
Scheme (**a**) and picture (**b**) of the experimental stand for testing the transfer characteristics of the fiber Bragg grating system.

**Figure 8:**
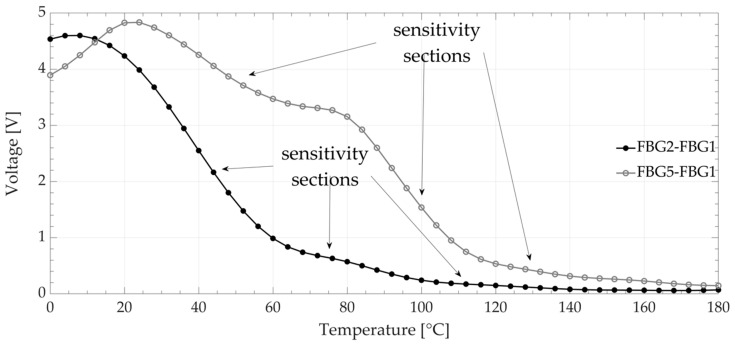
Experimental transfer characteristics.

Experimental verification was carried out using the appropriately selected fabricated structures mentioned earlier. The scheme of the interrogation system is shown in Figure 7. The FBG1 structure with the smallest *K* factor was used as the FBG sensor. Two FBGs with different side lobes were used as the FBG filter. The sensor structure was placed in a climate chamber whose temperature was changed from 0 to 180 degrees Celsius. Figure 8 shows a diagram and picture of the measuring system.

The operation of the FBG interrogation system was experimentally verified by temperature measurements inside a temperature chamber in which the sensor grating was placed. As a result of the experiment, the transition characteristics shown in Figure 8 were obtained.

Experimental verification performed for the FBG2-FBG1 and FBG5-FBG1 pairs confirmed the results of numerical calculations. The interrogation system processing curve had different slopes in various parts. For the first pair of fiber Bragg gratings (FBG2-FBG1), sensitivities of 0.0843, 0.0162, and 0.0036 (V/°C) were obtained, and sensitivities of 0.0379, 0.0822, and 0.0072 (V/°C) were obtained for the pair of gratings FBG1-FBG5. The temperature measurement range (5% cut off level) in the presented system also changed from 92 °C for the pair with smaller side lobes to 132 °C for the pair with larger side lobe levels (FBG5-FBG1).

## 4. Analysis of the Obtained Results and Discussion

Inverse apodization of fiber Bragg gratings results in high reflectivity of their side lobes. In consequence, the width of the transfer characteristic of interrogation systems with such an FBG is increased. Thus, the measurement range is also directly increased. For the fabricated structures containing side lobes, the measurement range increased from 0.583 to 1.2 nm. The process of writing FBG with a large spectral width without introducing chirp is difficult. FWHM greater than 1.5 nm is achievable for short structures less than 0.5 mm long. Writing such short FBGs with high reflectance is difficult because reducing the length of the structure also reduces its reflection coefficient. Chirped fiber Bragg gratings (CFBGs) are an alternative, but in many fabrication systems it was difficult to obtain such structures with high reflectance and low FWHM values until recently. Nowadays there are new techniques and possibilities to achieve CFBGs with high reflectance and low FWHM values [27,28,29]. Hence, their use in interrogation systems is problematic. CFBGs often have irregular reflection values in their spectral range. Another effect of the side lobes is a change in the sensitivity of interrogation systems in specific fragments of transfer characteristics. The height of the side lobes determines the maximum sensitivity that can be achieved. Changing the reflectivity value of the side lobes and the FWHM of the sensor grating will adjust the sensitivity of the interrogation system, and this also allows the measurement range to be controlled.

The spectral range of measurements with the use of matched filter systems is limited by the FWHM of the sensor and filter structures, as well as the spectral shift between them. The wavelength measurement range of commercially available interrogators is quite wide and reaches up to 100 nm [30]. In practice, the measurement of physical quantities such as temperature or strain is limited by the material properties of the optical fibers used to construct the sensor. The maximum strain of an optical fiber with inscribed FBG periodic structures used as a strain sensor is typically 6000 με. In the case of temperature measurement, the limitation of the measuring range is the temperature resistance of the fiber Bragg grating and the fiber coating. For an acrylic coating, the maximum operating temperature is about 85 °C, and for a polyamide coating it is already 300 °C. Interrogation systems with a matched filter and structures with high FWHM allow to extend the measurement range of the spectral shift to about 3 nm [31]. This limits the temperature measurement to the value of about 270 °C and to the strain measurement to the value of about 2500 με.

FBG matched filter systems allow to obtain a frequency response of about 500 kHz and a wavelength resolution of 0.3 pm [16]. Commercial integrators achieve a maximum frequency of 5 kHz and typically 1 kHz [30]. In our case, as a result of repeated measurements, we achieved a relative resolution of 0.5 × 10^−3^, which in the case of a measuring range of 1.2 nm means a resolution of 0.6 pm and a temperature measurement resolution of 0.1 °C in the range from 10 °C to 120 °C. The typical measurement resolution for a commercial interrogator is 0.5 pm [30]. Matched filter systems are also at least five times cheaper than commercially available interrogator systems.

Matched filters technique is dedicated rather to the interrogation of a single sensor. However, thanks to an appropriate demultiplexing system, it is possible to detect quantities measured on the basis of signals from many sensors located on the same optical fiber. In the case of AWG (arrayed waveguide grating) systems, conceptually similar to matched filters systems, even up to 128 gratings can be multiplexed [32]. It is worth to emphasize that in such systems the shape of the FBG sensor spectral characteristics does not exclude the use of grating with high side lobes. Thus, it is possible to build distributed sensors using high side lobes FBG. In turn, the possibility of extending the measuring range and the possibility of shaping and adjusting of slope of the sensor transfer characteristics are the great advantages of using such gratings. In the article we have presented that the widest measuring range and the possibility of shaping the sensitivity occur for pairs: high side lobes sensor and low side lobes filter or low side lobes sensor and high side lobes filter. These properties and the considerations about high side lobes FBG presented in the paper will also apply to e.g., AWG systems, since, in principle, AWGs are matched filter systems composed of Gaussian-shaped filters with low side lobes. It should be noted that widening the measuring range causes a corresponding broadening of the spectrum occupied by one sensor and reduces the number of sensors that can be placed on one optical fiber.

## 5. Conclusions

This article proposes the use of side lobes to extend the measurement range of an FBG sensor interrogated with a uniform FBG. An extension of the measurement range was obtained with a slight change in the shape of the transfer characteristics of the system. This solution maintains the simplicity of the measuring system and simplifies the FBG fabrication process due to the lack of chirp when writing structures in the optical fiber. The use of the proposed measuring system changes in an application in which the increased measurement range is not necessary allows one to use pairs of FBG structures that, due to easier adaptation to the required measurement range, are easier to fabricate.

It has been proven that proper selection of FBG parameters enables the design of an interrogation system with properties optimized for measuring the specific physical quantity in terms of matching the measurement range and changes in sensitivity of its individual parts.

## Figures and Tables

**Table 1:** Parameters of fiber Bragg gratings calculated for analysis.

Structure Number	a Coefficient	b Coefficient
1	2	0
2	3	0
3	5	0
4	10	0
5	20	0
6	20	0.1
7	20	0.2
8	20	0.3
9	20	0.4
10	20	0.5
11	20	0.6
12	20	0.7
13	20	0.8
14	20	0.9
15	20	1

**Table 2:** Parameters of fiber Bragg gratings fabricated for analysis.

	*K* [0..1]	Reflection [0..1]	FWHM [nm]
FBG1	0.0217	0.775	0.380
FBG2	0.1882	0.9165	0.664
FBG3	0.5615	0.5615	0.360
FBG4	0.7178	0.7178	0.372
FBG5	0.8260	0.8260	0.520
